# National trends and ecological factors of physical activity engagement among U.S youth before and during the COVID-19 pandemic: A cohort study from 2019 to 2021

**DOI:** 10.1186/s12889-024-19486-7

**Published:** 2024-07-17

**Authors:** Yuxin Zhu, Derwin K.C. Chan, Qianqian Pan, Ryan E. Rhodes, Sisi Tao

**Affiliations:** 1 Syns Institute of Educational Research, Hong Kong SAR, China; 2 Department of Early Childhood Education, The Education University of Hong Kong, 10 Lo Ping Rd, Tai Po, Hong Kong SAR China; 3 Centre for Research in Pedagogy and Practice (CRPP), Office of Education Research, National Institute of Education, Nanyang Technological University, Nanyang Ave, Singapore; 4 Behavioural Medicine Laboratory, School of Exercise Science, Physical and Health Education, University of Victoria, Victoria, Canada

**Keywords:** COVID-19, Physical activity, Children, Adolescents, Public health, Ecological factors, Recovery trends

## Abstract

**Background:**

This study aims to investigate the trends and ecological determinants of physical activity among U.S. children and adolescents during the 2019–2021 period, encompassing the COVID-19 pandemic’s onset and subsequent years.

**Methods:**

Utilizing data from the National Survey of Children’s Health over three years, this cohort study analyzed physical activity levels and ecological determinants among 82,068 participants aged 6–17. The sample included 36,133 children (44%) and 45,935 adolescents (56%), with variables assessed by caregiver reports.

**Results:**

The analysis revealed a significant decline in physical activity among children from 2019 to 2020, followed by a recovery in 2021, whereas adolescents showed a continued decrease without recovery. Over the study period, children were consistently more active than adolescents. Better health status, normal weight, less screen time, stronger peer relationships, higher parental involvement, better family resilience and greater school participation were consistently correlated with increased physical activity in both age groups. Sleep duration was a predictor of activity only in children, while mental health status was solely a predictor in adolescents. Neighborhood environment consistently predicted children’s activity levels but was a significant factor for adolescents only in 2020.

**Conclusions:**

These findings highlight the differing impacts of the pandemic on physical activity between children and adolescents, emphasizing the need for targeted public health interventions, particularly for adolescents whose activity levels have not recovered from the pandemic period. Age-specific physical activity interventions should consider sleep duration and neighborhood environmental factors when targeting children and mental health factors when focused on adolescents.

**Supplementary Information:**

The online version contains supplementary material available at 10.1186/s12889-024-19486-7.

## Introduction

Physical activity (PA) refers to any movement produced by skeletal muscles resulting in energy expenditure [1]. Participation in PA improves the overall growth and development of children and adolescents, including reducing the risk of mental health problems such as depression and anxiety, and chronic diseases such as cardiovascular disease, cancer, and diabetes [2, 3]. Despite the benefits of PA, data preceding the COVID-19 pandemic indicated that 53.6% of children and 81% of adolescents failed to meet the WHO-recommended threshold of 60 min of moderate-to-vigorous PA (MVPA) daily, regardless of family income level [4, 5]. The COVID-19 pandemic further compromised PA engagement in 2020, with a global decline of 17 min per day in children and adolescents’ MVPA, likely attributed to virus containment measures and enforced social distancing [6]. Although subsequent Canadian data suggest a partial rebound in MVPA in late 2020 [7], the extent to which PA levels have returned to pre-pandemic benchmarks in the post-pandemic context remains unclear.

PA is a result of an array of factors, encapsulated within an ecological framework that integrates biological and behavioral attributes (e.g., BMI and sleep patterns) [8, 9], individual characteristics (e.g., attitudes and self-efficacy) [10], social factors (e.g., family support and peer interactions) [11, 12], and environmental factors (e.g., access to public facilities) [13, 14]. This ecological framework, well-established in the literature [9], facilitates a comprehensive understanding of PA determinants, which is crucial for crafting effective multilevel interventions to encourage PA. While this framework has been used to analyze PA factors in both typical scenarios [9] and during the COVID-19 pandemic [15], comparative studies examining the change in these ecological factors during the pre- and post-pandemic restrictions phases are scarce. Furthermore, while individual level factors have been studied extensively, social and environmental influences have received comparatively less scrutiny [16, 17]. Similar to the role that etiological research plays in informing clinical treatments, a thorough analysis of the ecological factors influencing PA across these different periods is critical for enhancing coming public health initiatives and policy-making to improve PA levels.

In the current study, we analyzed a 3-year nationwide cohort (2019–2021) to examine trends in PA engagement among children and adolescents and to assess the evolution of ecological factors, especially social and environmental factors, associated with PA participation during this period. The study was guided by two research questions: (1) What were the trends in PA engagement among U.S. children and adolescents from 2019 to 2021? (2) How did the array of factors associated with PA participation evolve over this period? Informed by existing literature [7, 12, 18], we hypothesized that the reduction in PA observed during the COVID-19 pandemic has not fully reversed and that factors documented in the previous large-scale early pandemic studies would still be at play in the 2019 data. The exploration of the factors in 2020 and 2021 serves as an exploratory aim of this research.

## Method

### Ethics

This cohort study utilized data from the National Survey of Children’s Health (NSCH) spanning three years (2019–2021). Ethical approval was obtained for the original data collection [19]. This study used existing publicly available and deidentified data thus not qualifying as human subjects research. In conducting and reporting this study, we adhered to the Strengthening the Reporting of Observational Studies in Epidemiology (STROBE) guideline [20].

### Population, design and exposure

The methodology of the NSCH has been extensively detailed in prior literature [19]. In brief, the NSCH is a cross-sectional survey administered by the Census Bureau, collecting nationally representative data on the physical and mental health of U.S. youth ages 0–17. The survey employs an address-based sampling strategy to ensure representation from all 50 states and the District of Columbia. Households with at least one child under the age of 18 are eligible for participation. Within each selected household, one child is randomly chosen for inclusion in the survey. Data collection is primarily conducted via mail and web-based surveys, with telephone follow-ups for non-responding households. The survey collects data annually, spanning from June or July to January, and relies on responses from primary caregivers. Notably, the 2020 survey was conducted from June 2020 to January 2021, which was not interrupted by the COVID-19 pandemic.

For this study, we analyzed data from youth aged 6–17 years who did not have a serious motor disability, spanning the years 2019 to 2021. They were divided into two age groups aligned with previous literature: “children” for those aged 6–11 years, and “adolescents” for those aged 12–17 years [19, 21].

The exposure of this study was considered to be the experience of the COVID-19 pandemic and the associated public health restrictions. The year 2019 serves as the pre-pandemic cohort, 2020 corresponds to the cohort during the pandemic-related restrictions, and 2021 represents the cohort after the peak of the pandemic, meaning that while the initial crisis and highest levels of disruption had subsided, the situation was still influenced by the lingering effects and adjustments related to the pandemic.

### Primary outcome

The primary outcome of the study was MVPA. Caregivers rated the number of days a child participated in at least 60 min of exercise, sports, or physical activity during the past week using a four-point Likert scale: 0 days, 1–3 days, 4–6 days, and every day [22]. This measure aligns with the WHO’s MVPA guidelines for children and adolescents [23], thereby providing a standardized assessment of PA engagement.

### Predictor variables

Drawing from existing literature [9], predictors were categorized into four levels. *Biological and behavioral factors* included general health (1 item), overweight status (1 item), mental health status (1 item), sleep duration (1 item), and screen time (1 item). *Social predictors* encompassed parental involvement (1 item), family resilience (3 items), ease of making friends (1 item), and school activity (3 items). *Environmental predictors* were infrastructure quality (4 items) and neighborhood safety (5 items). *Demographic variables* were child age (1 item), gender (1 item), and parental education level (1 item). The following criteria for the inclusion of predictors were rigorously defined to ensure robust analysis: (a) consistent presence within the NSCH dataset throughout the three-year study period; (b) documented association with PA among the 6- to 17-year-old demographic in prior research; and (c) demonstration of satisfactory validity and reliability for factors comprising multiple items. A comprehensive list of variables and their psychometric performance is provided in Supplementary Table 1.

### Statistical analysis

Frequencies and mean levels of PA participation were presented as point estimates. Differences in PA participation over three years and between age groups were analyzed using the Mann-Whitney U test, which is a nonparametric test applied to ordinal data. It provides a Z-score and a two-tailed p-value, with a p-value of less than 0.05 considered to indicate a significant difference. Effect size Cohen’s d was calculated using Z-score and sample size according to the calculation suggested by Lenhard and Lenhard (2022) [24]. A value of 0.2 was considered a small, 0.5 a medium, and 0.8 a large effect size [25]. The Bonferroni correction was used to adjust for multiple comparisons, setting the significance threshold at *p* < .008, calculated as 0.05 divided by 6 comparisons (0.05/6 = 0.008). The analysis was performed using SPSS version 29. As the overall response rates were fairly consistent across the three years, and there was no strong or consistent evidence of nonresponse bias after survey weights were applied [26], missing data for this analysis were handled by listwise deletion, which is the default in SPSS.

Multigroup regression analyses (Group 1 = 2019, Group 2 = 2020, Group 3 = 2021) were employed to examine the evolution of multiple factors associated with PA participation from 2019 to 2021, separated for children and adolescents. PA was the dependent variable, predicted by the factors outlined in the Variables section. Single-item factors and multiple-item factors were modeled as observed and latent variables, respectively. For latent variables, the first item was fixed to 1.0 and all item error terms were freed for estimation. The weighted least square mean and variance adjusted (WLSMV) estimator was used to fit ordinal data, yielding standardized coefficients (β), 95% confidence intervals (95% CI), two-tailed *P* values, and R^2^ – indicative of effect size. Significance was set at *P* < .05 and 95% CI excluding zero. Stepwise modeling was used across the ecological spectrum to determine the R^2^ for each level of factors, beginning with demographic factors (Model 1), followed by the inclusion of biological/behavioral factors (Model 2), social factors (Model 3), culminating in a comprehensive model (Model 4) that incorporated all predictors. Only the comprehensive results of Model 4 and the R^2^ for each step are discussed in detail. Effect size R^2^ interpretations follow Cohen’s benchmarks: 0.01 (small), 0.09 (medium), and 0.25 (large). Model fit was appraised using the comparative fit index (CFI) > 0.90, the root mean square error of approximation (RMSEA) < 0.08, and the Tucker-Lewis index (TLI) > 0.90. Mplus version 8.3 facilitated the analyses, with the Full Information Maximum Likelihood approach addressing missing data.

## Results

A total of 82,068 participants were included in the study, comprising 36,133 children (44%) and 45,935 adolescents (56%). The annual distribution was 21,259 participants (26%) in 2019, 30,636 (37%) in 2020, and 30,173 (37%) in 2021. Among children, the median age was 9 years (IQR, 7–10), the mean age was 8.64 years (SD = 2.93), with 18,737 (52%) boys and 17,396 (48%) girls. The racial composition was 76.5% White, 7.4% Black or African American, 0.9% American Indian or Alaska Native, 5.7% Asian, 0.7% Native Hawaiian and Other Pacific Islander, and 8.8% identifying with two or more races. Among adolescents, the median age was 15 years (IQR, 13–16), the mean age was 14.67 years (SD = 2.91), with 23,865 (52%) boys and 22,070 (48%) girls. The racial breakdown was 77.5% White, 7.7% Black or African American, 1.1% American Indian or Alaska Native, 5.7% Asian, 0.8% Native Hawaiian and Other Pacific Islander, and 7.3% with two or more races. Table 1 provides detailed demographic descriptions of the participants.

**Table 1:** Child and family demographics

		Children, %			Adolescents, %		
Variables	Sample size (*N*)	2019	2020	2021	2019	2020	2021
Age group, y							
6–11 12–17	36,133 45,935	42.5 -	42.8 -	46.4 -	- 57.5	- 57.2	- 53.6
Sex							
Male Female	42,602 39,466	51.9 48.1	51.5 48.5	52.1 47.9	51.7 48.3	51.9 48.1	52.2 47.8
Race/ethnicity							
White Black or African American American Indian or Alaska Native Asian Native Hawaiian and other Pacific Islander Others	94,821 8,840 1,191 6,934 898 10,418	77.9 7.7 0.8 5.0 0.7 7.9	75.5 7.9 0.9 5.8 0.8 9.1	76.4 6.9 1.0 5.9 0.7 9.1	79.3 7.1 0.9 5.2 0.7 6.7	77.3 7.7 1.1 5.7 0.7 7.4	76.3 8.0 1.3 5.9 0.9 7.6
Primary household language							
English Spanish Other	113,446 4,615 5,041	93.4 3.1 3.5	92.5 3.6 3.9	91.6 4.0 4.4	94.0 3.0 3.0	92.7 3.9 3.4	91.6 4.5 4.0
Family structure of child’s household							
Two biological/adoptive parents, currently married Two biological/adoptive parents, not currently married Single mother Single father Grandparent household Other relation	84,415 7,297 17,981 5,523 3,463 1,187	68.7 7.7 14.6 4.2 3.9 1.0	67.9 5.9 16.5 5.3 3.4 1.0	69.9 6 15.3 5.1 3.0 0.9	70.2 5.3 15.4 4.8 3.1 1.1	68 4.2 18 5.9 2.8 1.0	66.1 4.5 19.1 5.8 3.1 1.4
Income level of child’s household							
Household income 0–99% FPL Household income 100–199% FPL Household income 200–399% FPL Household income 400% FPL or greater	15,802 20,267 37,834 49,919	11.7 17.1 31.5 39.7	13.0 17.8 30.9 38.3	13.2 16.9 30.3 39.6	10.3 15.7 31.5 42.4	12.1 16.4 29.9 41.5	13.3 16.2 30.3 40.2
Highest education of adults in child’s household							
Less than high school High school More than high school	3,198 16,117 103,787	2.2 13.6 84.2	2.6 13.5 83.9	2.5 13.6 83.9	2.7 13.3 84.0	3.1 14.3 82.6	3.5 14.5 82.0

Abbreviations: FPL, federal poverty level

### Trends in PA engagement among children and adolescents from 2019 to 2021

**Fig. 1:**
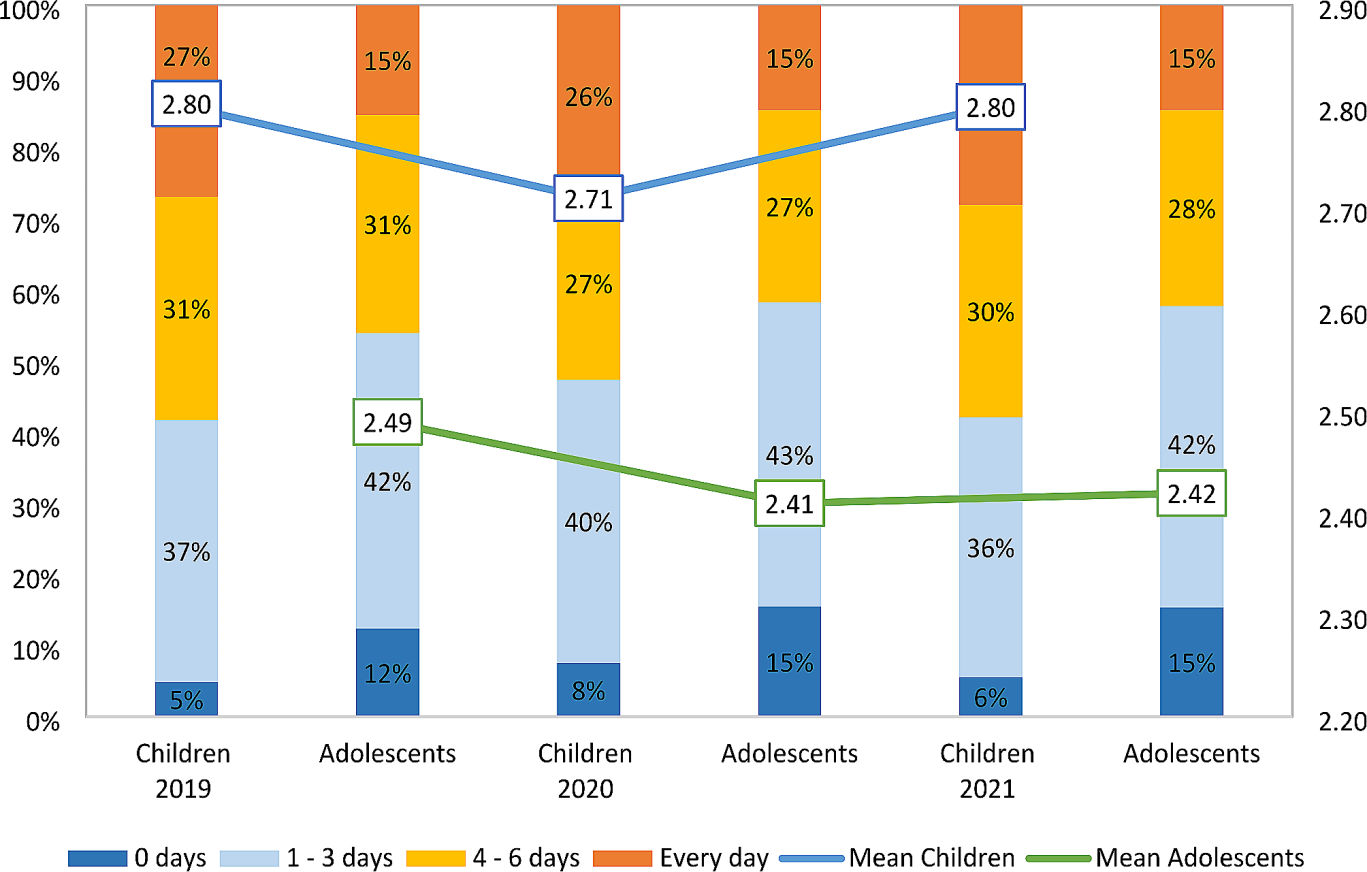
Frequencies and mean levels of PA participation across 2019 to 2021 in children and adolescents

Figure 1 depicts the three-year PA engagement trends for children and adolescents. Table 2 displays the Mann-Whitney U test outcomes, indicating a significant drop in children’s PA from 2019 to 2020 (Z = -8.39, *P* < .001, d = 0.10), with a subsequent rebound in 2021 (Z = -9.44, *P* < .001, d = 0.11). By 2021, PA reached the same levels as in 2019. Adolescents showed a significant PA decline from 2019 to 2020 (Z = -7.23, *P* < .001, d = 0.09), without a 2021 recovery, remaining decreased from 2019 levels (Z = -6.54, *P* < .001, d = 0.08). The effect size of change is considered small (all d < 0.2).

**Table 2:** Results of the Mann-Whitney U test (Z) for the physical activity participation

Year-to-year comparison					Age group comparison		
Years	Children		Adolescents		Children vs. Adolescents		
	Z	d	Z	d	Years	Z	d
2019 vs. 2020	8.39***	0.10	7.23***	0.09	2019	25.88***	0.36
2020 vs. 2021	-9.44***	0.11	-0.62	0.01	2020	28.59***	0.33
2019 vs. 2021	-0.21	0.00	6.54***	0.08	2021	36.98***	0.44

Across 2019, 2020, and 2021, children consistently engaged in more PA than adolescents (Z-scores of 25.88, 28.59, and 36.98, respectively; all *P* < .001). The effect sizes were small for the first two years (d = 0.33, 0.36) and approached moderate in 2021 (d = 0.44).

### Multiple factors associated with PA participation from 2019 to 2021

Multigroup model fits were satisfactory, with CFI = 0.90, RMSEA = 0.05, 90%CI [0.052, 0.053], TLI = 0.90 for children, and CFI = 0.91, RMSEA = 0.05, 90% CI [0.050, 0.051], TLI = 0.90 for adolescents. Detailed β values, 95% CIs, *P* values and R^2^ for each factor are presented in Table 3.

**Table 3:** Standardized coefficients, 95% confidence intervals, *P* values and *R*^*2*^ of factors associated with physical activity participation

	Children			Adolescents		
Factors	2019	2020	2021	2019	2020	2021
**Demographic variables**						
Parent education	-0.03** [-0.05, -0.02]	-0.01 [-0.03, 0.00]	-0.02 [-0.03, 0.00]	0.01 [-0.00, 0.02]	0.03*** [0.02, 0.04]	0.03*** [0.02, 0.04]
Youth gender	-0.08*** [-0.10, -0.06]	-0.08*** [-0.09, -0.06]	-0.09*** [-0.10, -0.07]	-0.15*** [-0.16, -0.13]	-0.12*** [-0.13, -0.11]	-0.13*** [-0.15, -0.12]
Youth age	-0.06*** [-0.07, -0.04]	-0.08*** [-0.10, 0.07]	-0.08*** [-0.10, -0.07]	-0.04*** [-0.06, -0.02]	-0.02* [-0.03, -0.00]	-0.03*** [-0.05, -0.02]
*R*^*2*^ (Model 1)	0.03***	0.04***	0.05***	0.03***	0.03***	0.03***
***Biological/Behavioral variables***						
General health	-0.11*** [-0.12, -0.09]	-0.09*** [-0.10, -0.07]	-0.08*** [-0.10, -0.07]	-0.12*** [-0.13, -0.10]	-0.12*** [-0.13, -0.11]	-0.12*** [-0.14, -0.11]
Overweight	-0.05*** [-0.07, -0.03]	-0.07*** [-0.09, -0.06]	-0.07*** [-0.08, -0.05]	-0.08*** [-0.10, -0.06]	-0.08*** [-0.09, -0.07]	-0.06*** [-0.08, -0.05]
Hour sleep	0.08*** [0.07, 0.10]	0.07*** [0.05, 0.09]	0.09*** [0.08, 0.11]	-0.01 [-0.02, 0.01]	-0.00 [-0.02, 0.01]	0.01 [0.00, 0.03]
Screen time	-0.17*** [-0.19, -0.15]	-0.21*** [-0.22, -0.19]	-0.18*** [-0.20, -0.17]	-0.19*** [-0.21, -0.18]	-0.20*** [-0.21, -0.19]	-0.18*** [-0.19, -0.16]
Mental health treatment	0.02 [0.00, 0.04]	0.01 [-0.00, 0.03]	0.02 [0.00, 0.03]	-0.05*** [-0.06, -0.03]	-0.05*** [-0.06, -0.04]	-0.05*** [-0.06, -0.03]
*R*^*2*^ (Model 2)	0.07***	0.09***	0.09***	0.14***	0.15***	0.14***
***Social variables***						
Make friend	-0.08*** [-0.10, -0.06]	-0.09*** [-0.10, -0.07]	-0.07*** [-0.08, -0.05]	-0.13*** [-0.14, -0.11]	-0.13*** [-0.14, -0.11]	-0.14*** [-0.15, -0.12]
Parental involvement	-0.10*** [-0.11, -0.08]	-0.11*** [-0.12, -0.10]	-0.09*** [-0.10, -0.08]	-0.11*** [-0.13, -0.10]	-0.13*** [-0.15, -0.12]	-0.14*** [-0.15, -0.13]
Family resilience	-0.07*** [-0.09, -0.04]	-0.08*** [-0.10, -0.06]	-0.08*** [-0.10, -0.06]	-0.09*** [-0.11, -0.07]	-0.05*** [-0.07, -0.04]	-0.06*** [-0.08, -0.04]
School participation	0.11*** [0.08, 0.14]	0.09*** [0.07, 0.11]	0.10*** [0.08, 0.12]	0.38*** [0.35, 0.41]	0.32*** [0.30, 0.34]	0.36*** [0.34, 0.38]
*R*^*2*^ (Model 3)	0.13***	0.17***	0.16***	0.34***	0.29***	0.31***
***Environmental variables***						
Infrastructure	0.02 [-0.00, 0.04]	-0.02 [-0.04, 0.00]	0.00 [-0.02, 0.02]	0.00 [-0.02, 0.02]	-0.00 [-0.02, 0.01]	0.00 [-0.01, 0.02]
Neighbourhood environment	-0.06*** [-0.08, -0.04]	-0.08*** [-0.10, -0.06]	-0.10*** [-0.11, -0.07]	-0.01 [-0.03, 0.02]	-0.05** [-0.06, -0.03]	-0.02 [-0.04, 0.00]
*R*^*2*^ (Model 4)	0.14***	0.18***	0.17***	0.34***	0.30***	0.31***

Note. * *P* < .05, ** *P* < .01, *** *P* < .001

Across 2019 to 2021, children with better health, normal weight, longer sleep duration, less screen time, stronger peer relationships, higher parental involvement, better family resilience, greater school participation, and living in friendly neighborhoods showed higher PA levels (β range: -0.11 to 0.11). Demographic factors accounted for 3–5% of the variance in PA over the three years, biological and behavioral factors for 4–5%, social factors for 6–8%, and environmental factors for 1%, indicating small to moderate effect sizes across the board.

Adolescents with better health, normal weight, less screen time, absence of mental health treatment, strong peer relationships, high parental involvement, robust family resilience, and active school engagement had consistently higher PA levels from 2019 to 2021 (β range: -0.20 to 0.38). A positive neighborhood environment was positively associated with PA only in 2020 (β = -0.05). Demographic factors accounted for 3% of the variance in PA over the three years, and environmental factors accounted for 0–1%, indicating small effect sizes. Biological and behavioral factors accounted for 11–12%, and social factors for 14–20% of the variance in PA over the three years, indicating medium to large effect sizes across the board.

## Discussion

The key findings of this national cohort study are twofold. First, a pandemic-associated decline in PA for both children and adolescents in 2020 compared to 2019 was observed, with only children’s PA recovering in 2021. Children were more active than adolescents across all years, with the gap widening post-pandemic restrictions. Furthermore, several factors—including better health status, normal weight, reduced screen time, stronger peer relationships, higher parental involvement, enhanced family resilience, and greater school participation—were consistently correlated with increased PA across both age groups. In contrast, sleep duration emerged as a predictor of PA exclusively among children, whereas mental health status was identified as a predictor only among adolescents. Additionally, while the neighborhood environment consistently influenced children’s PA levels, it was a significant factor for adolescents only in the year 2020.

Partially consistent with our hypothesis that the reduction in PA during the COVID-19 pandemic has not fully reversed, we found that MVPA levels in adolescents had not fully recovered, whereas children’s activity levels had rebounded by 2021. A previous review has documented a global reduction in PA during 2020. However, the patterns of PA recovery following the pandemic remain largely unexplored and seem to vary across different countries. To the best of our knowledge, only two studies utilizing national data have examined these post-pandemic recovery patterns, noting only partial recovery in Canada [27] and China [28]. Additionally, the continued and possibly increasing disparity in PA levels between children and adolescents post-pandemic underscores the urgent need for public health initiatives specifically designed to enhance PA, particularly among adolescents, who have not seen a recovery in their activity levels.

Interestingly, the percentage of individuals engaging in daily PA remained consistent across the three years for both age groups. Among children, those frequently active (4–6 days per week) saw a decrease in 2020, yet rebounded in 2021. Conversely, intermittent activity (1–3 days per week) and inactivity increased in 2020 but decreased in 2021. For adolescents, frequent PA patterns declined in 2020 without a subsequent recovery in 2021, leading to an increased percentage of intermittent activity and inactivity. This detailed analysis assists in pinpointing subgroups that may benefit from targeted interventions to boost their PA levels after the pandemic.

Both common and age- and time-specific factors associated with PA emerged in the multigroup analysis. Consistently, better health status, healthy weight, positive peer relationships, less screen time, more parental involvement, and good family resilience were associated with higher PA levels in both age groups throughout the study period. These results align with prior literature underscoring the significance of physical health, social support, and positive family dynamics in fostering regular PA [9, 29, 30]. In contrast to the well-documented relationship between other family factors and PA, family resilience only received increased attention after the pandemic as a protective factor for family emotional well-being in special scenarios such as COVID-19 [31]. Expanding previous findings, the positive association found in our study suggests that family resilience is also useful for maintaining PA levels in children and adolescents in the pandemic. These findings underscore the critical contribution of familial patterns and social dynamics to PA promotion, and are consistent with recent paradigms that prioritize family-oriented strategies for public health advances in pediatric populations [12, 32, 33].

Biological/behavioral and social factors were found to explain equal variance in children’s PA levels; however, for adolescents, social determinants showed a closer association, with a variance in PA engagement twice that attributed to biological/behavioral factors. This observation is corroborated by previous research indicating the significant impact of peers and friends on adolescent PA behaviors [34]. Moreover, in our analysis, the total variance in PA explained by the biological/behavioral and social factors was greater in adolescents than in children, suggesting that adolescent PA should be considered not only as a behavioral pattern but also as a social construct. These findings highlight the importance of incorporating social components within intervention strategies aimed at augmenting PA among adolescents.

In light of the differences in factors between children and adolescents, we speculate that adolescents may have exacerbated sedentary behaviors during the pandemic due to their greater reliance on digital devices for academic demands and peer interaction, as schools were intermittently closed due to the COVID-19 outbreaks between 2020 and 2021 [35–37]. This is supported by the NSCH data that adolescents reported more screen time than children the in 2021 dataset. Additionally, our analysis found that mental health status was a significant predictor of PA in adolescents but not in children. With worsening mental health commonly reported after the onset of the pandemic, it is plausible that poor mental health hindered adolescents from engaging in more PA [38]. These changes at the intrapersonal level likely reduced opportunities for offline PA engagement, partially explaining their lower PA levels in 2021. Interventions should therefore be sensitive to the dynamic social environments influencing youth behaviors and address the unique challenges posed by digital social interactions.

Consistent with previous research [27], a friendly neighborhood environment showed a modest positive association with children’s PA throughout the study period; however, this association was significant only for adolescents during the pandemic. The constraints imposed by pandemic-related restrictions and social distancing measures likely curtailed adolescents’ access to PA in school and other public spaces [6]. The observed shift underscores the heightened relevance of the neighborhood environment for PA in adolescents during this exceptional period and suggests the necessity to evaluate youth PA within the context of broader situational changes.

### Implications

Given the persistently low levels of PA among adolescents after the pandemic, it is crucial for future research to continuously monitor their PA levels to assess potential rebound. Additionally, nationwide, age-specific interventions are essential to enhance PA participation and prevent further declines.

To effectively design these interventions, leveraging insights from the ecological changes in factors predicting PA is key. For both children and adolescents, strategies focusing on health and weight management, such as nutritional education and regular health check-ups, could be beneficial. Parental involvement is also critical, with an emphasis on promoting family-oriented PA for children and activities that engage peers for adolescents. School-based initiatives should aim to strengthen sports programs, with a special focus on team sports and physical education that are age-appropriate. The positive impact of intramural sports, as opposed to interscholastic sports, on increasing PA could be considered in these programs [39].

Tailored approaches appear necessary for each age group. For children, ensuring adequate sleep and fostering a friendly neighborhood environment can be particularly beneficial. In contrast, for adolescents, integrating mental health support with PA initiatives, such as programs that combine exercise with stress management, may prove effective [40, 41]. These targeted strategies can help address the unique needs and challenges faced by different age groups in maintaining an active lifestyle.

### Strengths and limitations

Our study has several strengths. First, the dataset used in our study includes a large and nationally representative which enhances the generalizability of the results, as it covered a wide age range of children and adolescents. Second, our report about the trends of PA before, during, and after the onset of COVID-19 may provide a comprehensive view of how the pandemic has affected PA over time. Third, our findings regarding the ecological changes in PA-related factors over three years may offer valuable insights into the various influences on PA during this period. These strengths ensure that the study captures a detailed and accurate picture of PA trends and determinants, making the findings robust and relevant for informing targeted intervention programs.

This study has its limitations. Firstly, the sample is confined to the U.S., limiting the generalizability of the findings to a global context [42]. Second, the study relies on parent-reported PA, which, compared to objective measurements using devices, may be subject to recall and social desirability biases. Furthermore, some variables in this study were measured using single-item questions, which could also lead to measurement bias. Finally, psychological factors such as self-efficacy and motivation were not included in the analysis given their role is well established [9]. These limitations suggest that while the study provides valuable insights, its findings should be interpreted with caution, especially when considering their applicability to populations outside the U.S.

## Conclusion

The study underscores the profound impact of the COVID-19 pandemic on PA levels in U.S. children and adolescents. It shows a marked decline in PA in 2020, followed by a recovery in 2021 for children, but a continued decline in adolescents. These findings highlight the critical need for interventions tailored to different age groups. Such interventions should take into account a range of ecological factors at the demographic, biological/behavioral, social and environmental levels to effectively promote PA, with a particular focus on the uniquely affected adolescent population.

### Electronic supplementary material

Below is the link to the electronic supplementary material.

Supplementary Material 1

## Data Availability

Data supporting the results of this study have been published on the National Survey of Children’s Health (NSCH) website (https://www.childhealthdata.org/learn-about-the-nsch/NSCH).

## Abbreviations

PA: Physical Activity
MVPA: Moderate-to-Vigorous Physical Activity
BMI: Body Mass Index
NSCH: National Survey of Children’s Health
STROBE: Strengthening the Reporting of Observational Studies in Epidemiology
WLSMV: Weighted Least Square Mean and Variance Adjusted
RMSEA: Root Mean Square Error of Approximation
TLI: Tucker-Lewis Index
