# Supplementary material for: National trends and ecological factors of physical activity engagement among U.S youth before and during the COVID-19 pandemic: A cohort study from 2019 to 2021
Source: BMC Public Health. 2024 Jul 17;24:1923. doi: 10.1186/s12889-024-19486-7 (PMC11256660; doi:10.1186/s12889-024-19486-7)
Supplement: Supplementary file 1 — Supplementary Material 1 [file 12889_2024_19486_MOESM1_ESM.docx]

**Supplementary Table 1**. List of ecological variables

| **Levels** | **Variable names/labels** | **Questions** | **Rating scales** |
| --- | --- | --- | --- |
| Biological and behavioral factors | General health  (K2Q01) | In general, how would you describe this child’s health? | 1=excellent … 5 = poor |
|  | Overweight status (BMICLASS) | How much does this child CURRENTLY weigh? | 1=less than the 5^th^ … 4 = greater than the 95^th^ |
|  | Mental health status (nom18MHlth) | Children ages 3-17 years reported by their parents to have been diagnosed by a health care provider with a mental/behavioral condition (depression,  anxiety problems, or behavioral or conduct problems) | 1 = no; 2 = yes |
|  | Sleep duration (HOURSLEEP) | During the past week, how many hours of sleep did this child get during an average day (count both nighttime sleep and naps) (0-5 years)/on most weeknights (6-17 years), age 4 months-17 years? | 1 = children sleeps less than recommended age-appropriate hours; 2 = children sleeps recommended age-appropriate hours |
|  | Screen time (SCREENTIME) | ON MOST WEEKDAYS, about how much time does this child usually spend in front of a TV, computer, cellphone or other electronic device watching programs, playing games, accessing the internet or using social media? | 1 = less than 1 hour … 5 = 4 or more hours |
| Social factors | Parental involvement (K7Q33) | During the past 12 months, how often did you attend events or activities  that this child participated in, age 6-17 years? | 1 = always … 5 = never |
|  | Family resilience (TALKABOUT)  (WKTOSOLVE)  (HOPEFUL) | When your family faces problems, how often are you likely to do each of the following?  Talk together about what to do.  Work together to solve our problems.  Stay hopeful even in difficult times. | 1 = all of the time … 4 = no of the time |
|  | Make friend (MAKEFRIEND) | Compared to other children his or her age, how much difficulty does this child have making or keeping friends? | 1 = no difficulty … 3 = a lot of difficulty |
|  | School participation  (K7Q30) (K7Q31) (K7Q32) | DURING THE PAST 12 MONTHS, did this child participate in:  A sports team or did he or she take sports lessons after school or on weekends?  Any clubs or organizations after school or on weekends?  Any other organized activities or lessons, such as music, dance, language, or other arts? | 1 = no; 2 = yes |
| Environmental factors | Infrastructure  (K10Q11) (K10Q12) (K10Q13) | In your neighbourhood, is/are there:  Sidewalks or walking paths?  A park or playground?  A recreation center, community center, or boys’ and girls’ club? | 1=yes; 2=no |
|  | Neighborhood safety (K10Q30) (K10Q31) (K10Q40_R) (GOFORHELP) | To what extent do you agree with these statements about your neighbourhood or community?  People in this neighbourhood help each other out.  We watch out for each other’s children in this neighbourhood.  This child is safe in our neighbourhood.  When we encounter difficulties, we know where to go for help in our community. | 1= definitely agree … 4= definitely disagree |
| Demographic factors | Youth age | What is the child’s age? | (number of age) |
|  | Youth gender | What is the child’s sex? | 1 = male; 2 = female |
|  | Parental education level | What is the highest education of adult in this child’s household? | 1 = less than high school education; 4 = college degree or higher |
